# Supplementary material for: Conditional chemoconnectomics (cCCTomics) as a strategy for efficient and conditional targeting of chemical transmission
Source: eLife. 2024 Apr 30;12:RP91927. doi: 10.7554/eLife.91927 (PMC11060718; doi:10.7554/eLife.91927)
Supplement: Figure 3—source data 3. [file elife-91927-fig3-data3.zip › Figure 3 source data3 part 1.zip!/Figure 3-Source data 3-part1/1063-13-CRISPResso_Report_8cz_fsdr.zip!/CRISPResso_on_1063_16/1c.Alignment_barplot.pdf]

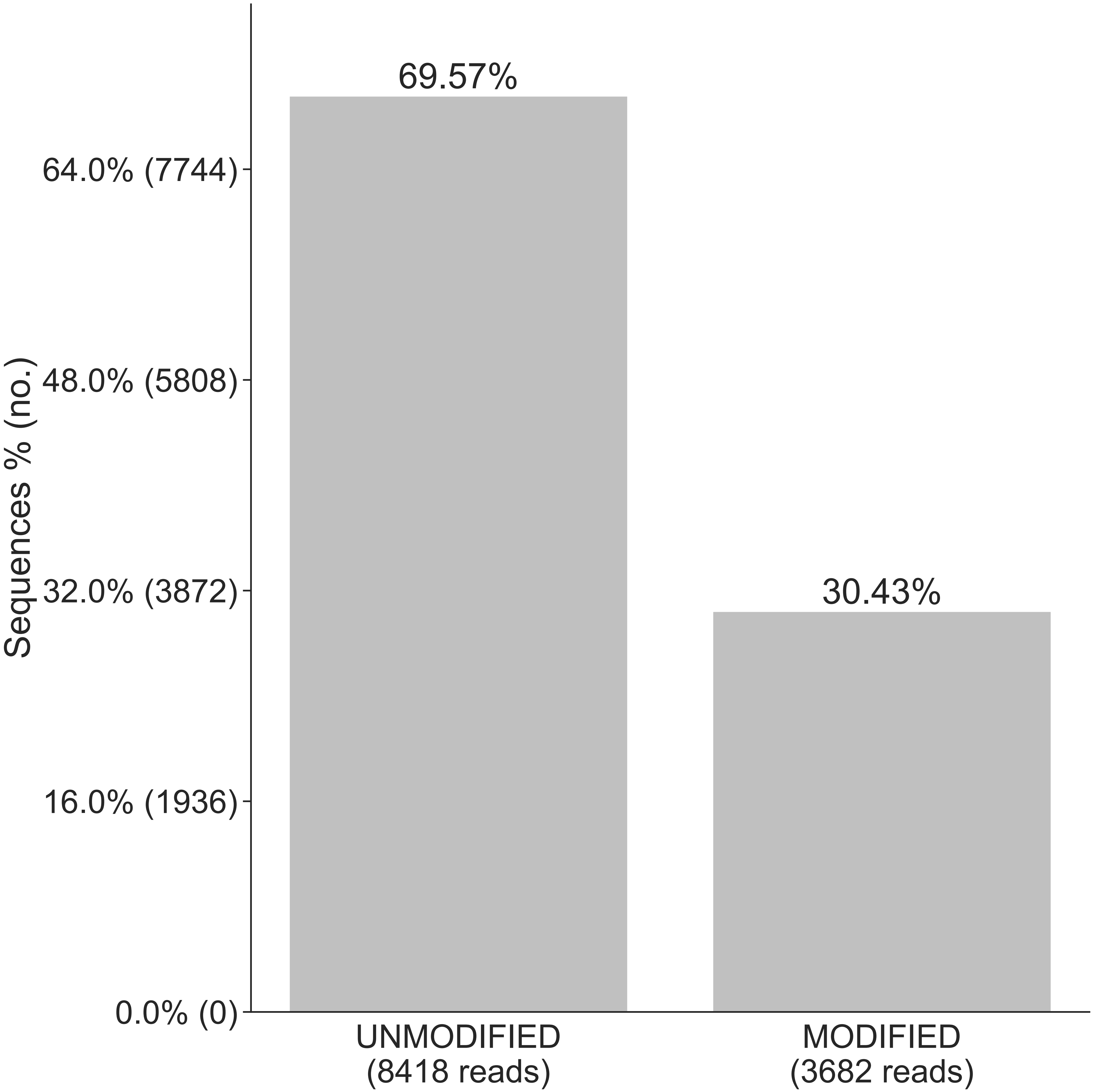

69.57%
30.43%
Sequences % (no.)
64.0% (7744)
48.0% (5808)
32.0% (3872)
16.0% (1936)
0.0% (0)
UNMODIFIED
(8418 reads)
MODIFIED
(3682 reads)
